# Composition analysis of fractions of extracellular polymeric substances from an activated sludge culture and identification of dominant forces affecting microbial aggregation

**DOI:** 10.1038/srep28391

**Published:** 2016-06-17

**Authors:** Xuan Guo, Xu Wang, Junxin Liu

**Affiliations:** 1 Research Center for Eco-Environmental Sciences, Chinese Academy of Sciences, Beijing 100085, China; 2 University of Chinese Academy of Sciences, Beijing 100049, China; 3 State Key Laboratory of Aquatic Environmental Chemistry, Research Center for Eco-Environmental Sciences, Chinese Academy of Sciences, Beijing 100085, China

## Abstract

Extracellular polymeric substances (EPS) appear to play a critical role in the formation of bioaggregates, such as sludge flocs, in activated sludge processes. Here, we systematically investigated the composition and chemical structure of various EPS fractions excreted from an activated sludge culture using multi-analysis techniques to examine the ability of the sludge to aggregate. Chemical analysis was used with a three-dimensional excitation emission matrix and Fourier transform infrared spectroscopy, applying inter-particle forces theory. The combined findings revealed that hydrophobic groups, especially protein-related N–H, were present in a greater proportion in tightly bound EPS (TB-EPS). This result, which explained the specificity of TB-EPS in the chemical structure, was consistent with data indicating that TB-EPS contained a large amount of protein-like substances (86.7 mg/g of mixed liquor volatile suspended solids, 39.7% of the total EPS). Subsequently, a novel experimental procedure was developed to pinpoint key inter-particle forces in sludge aggregation. The result revealed that hydrogen bonds are the predominant triggers that promote sludge aggregation. This comprehensive analysis indicated that hydrophobic proteins in TB-EPS are responsible for the critical role played by hydrogen bonds in sludge formation. Our findings highlight the need to elucidate the mechanisms of TB-EPS-mediated flocculation in future efforts.

The conventional activated sludge process (ASP) and its alternatives are extensively applied in wastewater treatment worldwide. In the ASP, microorganisms form concentrated bioaggregates, enabling efficient wastewater treatment^1^. However, serious challenges remain that may limit potential improvements in the ASP. One of the most frequent issues is sludge bulking, which can be triggered by deflocculation, and which significantly hinders the downstream settling and dewatering processes^2^. Extracellular polymeric substances (EPS), which are released by microbes, have been reported to play a critical role in the formation of bioaggregates, such as sludge flocs, in the ASP^3^. EPS are essential building blocks of aggregates and account for nearly 80% of the mass of activated sludge^4^. Therefore, deciphering the mechanisms of microbial aggregation related to EPS formation is crucial in order to develop effective strategies to counter sludge bulking.

EPS, which are present both outside microbial cells and within bioaggregates, are composed of proteins, polysaccharides, and humic substances, among other substances^5^. Two types of EPS are known and may be separated by centrifugation: EPS remaining in the supernatant are soluble (S-EPS), and those that form biopellets constitute bound EPS^6^,^7^. Bound EPS may be described by a two-layer model^8^, which consists of tightly bound EPS (TB-EPS, the inner layer of which has a definite shape and is bound tightly and stably with the cell surface) and loosely bound EPS (LB-EPS, the outer layer of which is loose and dispersible, without an obvious edge).

Although considerable research on EPS-related topics has been conducted, reports describing the effect of EPS structure on bioflocculation provide conflicting data. A few studies have demonstrated that greater EPS production markedly increases sludge aggregation^9^,^10^, whereas other studies have found that the flocculation ability of sludge flocs decreases with augmented EPS generation^3^,^11^. Considering that there are differences and variations in EPS extraction methods^12^, and that the majority of previous results used correlation analysis rather than direct evidence^13^,^14^, it is not surprising that the literature contains conflicting information. Furthermore, some types of EPS, such as TB-EPS, are independent of the formation of bio-aggregates^15^. Therefore, the dominant variables determining sludge aggregation and the corresponding structure and composition of EPS in sludge flocs remain to be elucidated.

Cell–aggregate interaction to form activated sludge flocs is a dynamic process that results from physiochemical activities originating in the EPS-mediated microbial behaviors^16^. Although previous work has attempted to illustrate ion interactions and hydrogen bonds among other mechanisms for cell aggregation^7^,^14^,^17^,^18^, further efforts are required to investigate the differences in cellular physiochemical interactions that are relevant to the composition, chemical groups, and structure of sludge EPS, as well as to determine potential links between specific EPS components and microbial aggregation. Elucidation of these topics will greatly enhance our understanding of microbial aggregation.

The present study investigates the hypothesis that microbial aggregation of activated sludge is controlled not only by the total amount of EPS, but also by the chemical functionalities and structure of EPS. EPS were extracted from an activated sludge culture and the composition and chemical structure of each EPS fraction were further characterized using a three-dimensional excitation emission matrix (3D-EEM) and Fourier transform infrared spectroscopy (FTIR), among other techniques. In addition, an *in situ* trial was performed to examine the role of the EPS fractions in sludge aggregation.

## Results and Discussion

### Characterization of EPS from activated sludge

Activated sludge–derived EPS from an A^2^/O-driven wastewater treatment plant were characterized using chemical analysis, 3D-EEM, and FTIR measurements. The results of these analyses are described here.

#### Chemical analysis

Extracted EPS content and floc composition are presented in Table 1. Specifically, the total EPS contained in the activated sludge flocs included about 218.40 mg/g of mixed liquor volatile suspended solids, in which the average proportions of individual S-EPS, LB-EPS, and TB-EPS accounted for 7.4%, 22.6%, and 70.0% of the total EPS, respectively, with TB-EPS representing the predominant fraction (S-:LB-:TB-EPS ratio = 0.1:0.3:1). EPS composition varies at different stages or operational conditions in the same activated sludge sample, but TB-EPS has been reported as the main fraction in other studies as well^15^.

It is evident that proteins, polysaccharides, and humic substances are the major constituents of EPS^19^. In the present study, proteins, polysaccharides, and humic substances constituted 50.2%, 40.0%, and 9.8%, respectively, of the total EPS. As suggested in previous reports, the predominance of proteins in EPS may be attributed to the presence of a large quantity of exoenzymes in the EPS matrix, arising from the ready degradation and update of biodegradable organics in the ASP^20^. In addition, differences were further highlighted when the proportions of proteins, polysaccharides, and humic substances in the S-EPS, LB-EPS, and TB-EPS fractions (see Table 1) were analyzed. In both S-EPS and LB-EPS fractions, polysaccharides, which composed 52.0–69.6% of the total, were the main fraction, followed by proteins, which composed 27.3–37.7%. Humic substances accounted for a small proportion of the EPS, 3.7–10.3%. In contrast, TB-EPS contained a much higher quantity of proteins (HS:PS:PN ratio = 0.2:0.6:1).

#### 3D-EEM assays

**Figure 1:**
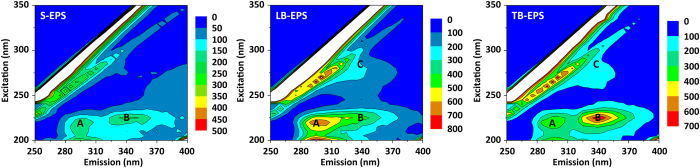
Three-dimensional excitation emission matrix (3D-EEM) fluorescence spectra of soluble extracellular polymeric substances (S-EPS), loosely bound extracellular polymeric substances (LB-EPS), and tightly bound extracellular polymeric substances (TB-EPS).

The 3D-EEM fluorescence spectra of each EPS fraction from the activated sludge sample are shown in Fig. 1. The fluorescence peak positions and intensity in the 3D-EEM spectra of the different EPS fractions were identified according to previous studies^21^,^22^. In the present work, two distinct peaks were clearly observed in the 3D-EEM fluorescence spectra of S-EPS, and three distinct peaks were identified in the spectra of both LB-EPS and TB-EPS. Specifically, Peak A was observed at the excitation/emission wavelengths (Ex/Em) of nearly 230/295 nm, whereas Peak B was identified at the Ex/Em of about 230/340 nm, and Peak C was found at the Ex/Em of 270/340 nm. According to the literature, Peaks A, B, and C are described as the tryptophan proteins peak, tyrosine amino acids peak, and humus-like substances peak, respectively^14^,^23^,^24^. 3D-EEM fluorescence spectroscopy has been extensively applied for indirect quantitative analysis based on fluorescence variables such as peak intensity and different peak intensity ratios. As further shown in Fig. 1, the intensities of Peak A and Peak B were significantly higher than those of other fluorescence peaks, a result that was consistent with the greater fraction of proteins detected in the EPS (see Table 1). This result highlighted that protein is the predominant constituent of EPS and implied the significant existence of proteins in the activated sludge floc structure, in agreement with previous findings^14^. Despite this, the different fluorescent depictions of the three EPS fractions suggested that the compositions of S-EPS, LB-EPS, and TB-EPS vary. Specifically, Peak C appeared only in the spectra of LB-EPS and TB-EPS (Fig. 1). Obviously, the components of S-EPS were less diverse than those of either LB-EPS or TB-EPS, and the content of humus-like substances in bound EPS was higher than that in soluble EPS. These findings further illustrated that the two different types of EPS (soluble and bound) exist within the sludge flocs, and varying aggregation mechanisms of flocs and microcolonies may lead to different compositions of soluble and bound EPS.

#### FTIR analysis

**Figure 2:**
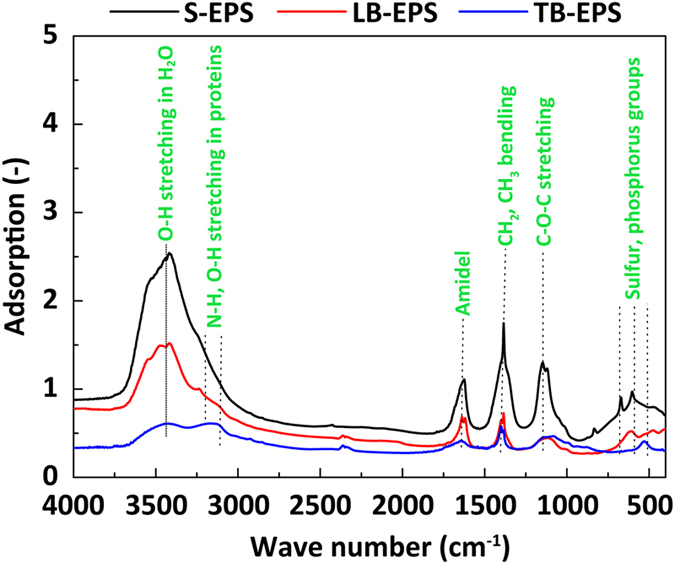
Fourier transform infrared spectroscopy (FTIR) spectra of soluble extracellular polymeric substances (S-EPS), loosely bound extracellular polymeric substances (LB-EPS), and tightly bound extracellular polymeric substances (TB-EPS).

FTIR analysis was carried out to investigate the chemical structures of the various activated sludge EPS fractions. Data are depicted in Fig. 2. The positions and number of FTIR peaks for the EPS fractions initially appeared to be quite close, implying that the types of chemical groups in these fractions were similar. Several strong frequency bands associated with proteins and polysaccharides were readily observed in the EPS components, among which were the stretching vibration of N-H and O-H (3,500–3,100 cm^−1^), the stretching vibration of C=O (1,635 cm^−1^), the bending vibration of CH_2_ (1,384 cm^−1^), and the C–O–C stretching vibration of polysaccharides (1,153–1,072 cm^−1^); these findings were consistent with previous reports^10^,^25^. However, a much closer inspection of the intensity of the respective peaks illustrated that the relative contents of chemical groups were distinctly different. First, the peak at nearly 1,080 cm^−1^ was assigned to the vibration absorption peak of hydroxyl in polysaccharides^26^, which was much stronger in bound EPS than in soluble EPS. Secondly, the band at 1,153 cm^−1^ was associated with the C–O–C bending in polysaccharides, which was almost invisible in bound EPS but obvious in soluble EPS. Third, the bands at 3,200–3,000 cm^−1^ were attributed to the N–H and O–H stretching vibrations, where a relatively wider and higher intensity pattern was clearly observed for TB-EPS than for other EPS, as shown in Fig. 2. Finally, the peak at 3,100 cm^−1^ corresponded to N-H and O-H in tyrosine-like substances and was much smaller for S-EPS and LB-EPS than for TB-EPS. This observation, which was consistent with the results of the FTIR analysis, strongly indicated that the EPS fractions consisted of different components and suggested that a high proportion of hydrophobic components existed in TB-EPS because TB-EPS contained the majority of proteins (see Table 1).

### Identification of key inter-particle forces in sludge aggregation

Inter-particle forces, such as ion interactions and hydrogen bonds, are hypothesized to interfere with cell–aggregate interactions, thereby influencing the formation of sludge flocs. However, few studies have focused on identification of inter-particle forces in sludge aggregation. A typical method for mechanistic exploration of microbial flocculants was used in this work (see the Methods section for details), utilizing ethylene diamine tetraacetate (EDTA) and urea to pinpoint ion interactions and hydrogen bonds, respectively.

**Figure 3:**
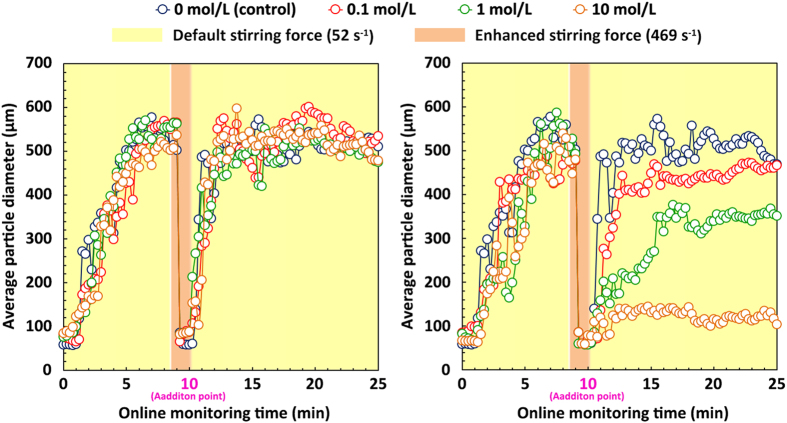
Effects of inter-particle forces on sludge aggregation. **(a)** Ethylene diamine tetraacetate (EDTA) was added at 4 different doses to 4 ideal reactors for 15 min to explore the potential role of ionic interactions in sludge aggregation. **(b)** Urea was added at varying doses to 4 additional reactors for 15 min to test the potential link between hydrogen bonds and sludge aggregation.

Following the start of floc formation (Fig. 3), floc size increased with stirring time in all the samples tested and finally reached a stable floc diameter of 500–600 μm after 5 min (*p* > 0.05). After 8.5 min, the stirring speed was increased to 1300 rpm (G = 469 s^−1^), which resulted in the rapid breakdown of the sludge flocs and reduction of the average floc diameter to less than 100 μm within 1.5 min. We determined that greater shear force effectively broke the sludge flocs into smaller clusters.

To further test the consequences of ion interactions and hydrogen bonds, various doses of EDTA (Fig. 3a) and urea (Fig. 3b) were added to the reactors at the end of 10 min. An increase in EDTA concentration did not affect cluster aggregation over time compared with the control (*p* > 0.05); the small clusters aggregated and grew to the size of the original sludge flocs (500–600 μm). Therefore, the addition of EDTA did not reduce the aggregation ability of the sludge clusters. EDTA is one of the most significant reagents for complexometric determination of metal ions in solution^27^, as it can form strong complexes with most metal ions and thus interrupt ion interactions. Activated sludge contains many functional groups, such as carboxyl, hydroxyl, and amino acid groups, that can be easily bridged with cations, such as Ca^2+^, Al^3+^, Fe^3+^ and Mg^2+^, in the sludge matrix^12^. Ion interactions may be a potential trigger that induces bioflocculation^28^. However, the present finding indicated that ion interaction was not the dominant force that triggers sludge aggregation, because the aggregation was not significantly affected by the addition of EDTA.

In contrast, incremental addition of urea significantly reduced sludge aggregation over time (*p* < 0.05). The minimum average floc diameter (~100 μm), which was approximately 4-fold lower than that of the control (Fig. 3b), was stably achieved at a urea dose of 10 mol/L, after 5 min of stirring. Urea can destroy hydrogen bonds in microbial flocculants and thus reduce bio-aggregation efficiency^29^,^30^,^31^. This result suggested that the formation of hydrogen bond is the major trigger that promotes sludge floc aggregation. Further efforts should be devoted to elucidating and proving the relevant mechanisms using multidisciplinary analysis techniques.

### Roles of various EPS fractions in sludge aggregation

**Figure 4:**
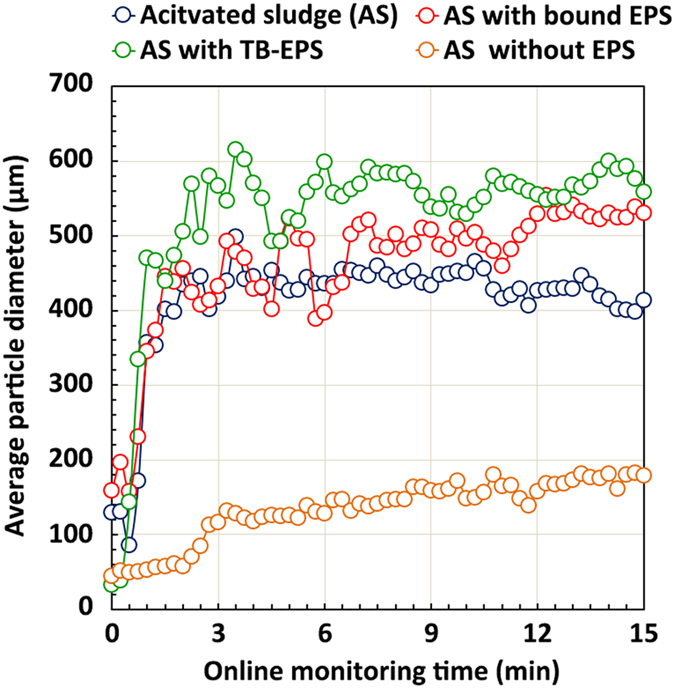
Effects of the components of various extracellular polymeric substances (EPS) on sludge aggregation performance, represented by changes in average particle diameter of flocs over time.

The small-angle laser light-scattering (SALS) technique was used to explore the effects of the EPS fractions on sludge aggregation *in situ*, based on size. The results are shown in Fig. 4. The sludge clusters from which EPS was not extracted were capable of aggregating with other clusters over a short period of time (within 1–2 min) and achieved stability at the end of 15 min with an average aggregate diameter of approximately 450 μm (blue points). Interestingly, the aggregation performance of sludge flocs was significantly enhanced by the simultaneous elimination of S-EPS and LB-EPS (green points), as shown by an increase in the floc diameter to 500–600 μm, whereas the floc diameter sharply fell to below 200 μm when TB-EPS was also extracted from the sludge flocs (orange points).

**Figure 5:**
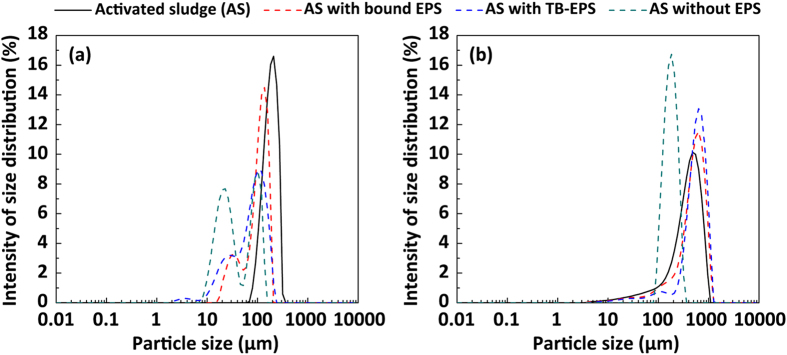
Roles of various fractions of soluble extracellular polymeric substances (EPS) in the sludge aggregation process, reflected by changes in the size distribution profiles of the activated sludge particles, whose diameters were determined by small-angle laser light scattering, before **(a)** and after **(b)** the expected aggregation.

Size distribution profiles of the various sludge flocs before aggregation (time = 0 min) and after aggregation (time = 15 min) are also presented. Before aggregation (Fig. 5a), the diameter of the original sludge flocs declined steeply (from 129 μm to 82 μm) following the removal of both S-EPS and LB-EPS, at the same time that more sludge flocs with a diameter in the range of 10–60 μm were noted. After the simultaneous removal of S-EPS and LB-EPS and after 15 min of aggregation, the sludge flocs had expanded in size to 400–1,000 μm, suggesting that larger flocs were formed during aggregation without the outer layer EPS. These results indicate that the presence of S-EPS and LB-EPS hinders the aggregation of sludge flocs to some extent, and that TB-EPS plays a critical role in sludge aggregation.

As shown previously, TB-EPS consisted of a greater proportion of proteins and a smaller percentage of polysaccharides than did the S-EPS and LB-EPS fractions. As the predominant component of TB-EPS, proteins play a more essential role then do polysaccharides in the aggregation of activated sludge^10^,^26^. This understanding is supported by previous reports that microbial aggregates are deflocculated after removal of surface proteins^32^, whereas the flocculation ability of activated sludge increases with augmentation of protein content in the flocs^19^.

### Implications of this work

Recent microbial aggregation studies have drawn much attention to the bridging mechanisms of ionic and hydrogen bonds^33^,^34^,^35^. The present findings suggest that hydrogen bonds, rather than ionic interactions, are responsible for cell–aggregate interactions; in other words, they trigger sludge floc formation (Fig. 3b). Nevertheless, hydrophobic interactions are thought to represent long-range forces that contribute significantly to activated sludge aggregation^14^. Hydrophobic interaction, which is essential for microbial aggregation, occurs in numerous experimental phenomena: microbial flocculation ability is positively correlated with microbe-surface hydrophobicity^9^. Activated sludge flocs, which adsorb organic matter with low solubility in water through hydrogen bonds^36^, have been demonstrated (via spectroscopic analysis) to comprise hydrophobic interaction regions^37^. The high aggregation ability of sludge pellets with TB-EPS (Figs 4 and 5) is attributed to their high hydrophobicity levels, which result from the composition and chemical structure of TB-EPS derived from activated sludge.

The present investigation of chemical structure via 3D-EEM and FTIR spectroscopy revealed that a greater proportion of hydrophobic groups, especially protein-related N–H, was present in activated sludge–derived TB-EPS (Figs 1 and 2). This finding, which explains the specificity of TB-EPS in the chemical structure, is consistent with data indicating that TB-EPS contains a high amount of protein-like substances (Table 1). This result further corresponds with those of other studies showing that proteins in EPS play a key role in the surface hydrophobicity of sludge^38^. Proteins may be hydrophobic due to the presence of hydrophobic *R* groups in amino acids, and their hydrophobicity is determined by their constituents and structure. Therefore, future efforts must be devoted to elucidating the key characteristics, in particular the amino acid composition and secondary structure, of proteins in activated sludge–derived TB-EPS.

In addition, various EPS (e.g., in biofilms, soils, and marine sediments) have been found to contain extracellular deoxyribonucleic acids (e-DNA), in particularly large amounts during the early stages of biofilm growth^39^,^40^, although the amount produced can vary even between closely related species^41^. For example, e-DNA produced by some bacteria, such as *Pseudomonas aeruginosa*, function as a cell-to-cell interconnecting matrix component in biofilms^42^. In addition, it has been reported that the released e-DNA, as well as its transformation. is part of a biofilm life cycle and functions to stabilize the biofilm structure^43^. Recent literature further reported that e-DNA was a major component of EPS matrix from sludge that was purely cultured by *Staphylococcus aureus*^41^; however, e-DNA to date have been found to be the minority of EPS extracted from real-life activated sludge cultures^44^. Consequently, the existence of e-DNA in the EPS matrix and its potential role on sludge floc aggregation were not explored in this work. However, future efforts should explore the role of e-DNA in EPS-mediated aggregation of real-life activated sludge.

## Conclusions

The key characteristics of EPS fractions, namely their composition, chemical groups, and structure, were examined in an activated sludge culture. The large amount of hydrophobic proteins in the TB-EPS fraction was responsible for the dominant role of hydrogen bonds in sludge aggregation. In addition to elucidating critical links between TB-EPS and sludge aggregation, the present findings indicate that future research efforts must determine the main characteristics of proteins in the TB-EPS fraction. In particular, their amino-acid composition and secondary structure should be elucidated to improve our understanding of TB-EPS mediated flocculation.

## Methods

### Activated sludge sample, EPS extraction, and chemical analysis

An activated sludge sample was derived from a secondary clarifier treating municipal effluent (6.0 × 10^5^ m^3^/d) from an anaerobic/anoxic/oxic (A^2^/O) wastewater treatment plant in Beijing, China. After on-site sampling, the sludge sample was delivered immediately to the laboratory within 2 h, filtered through a 1.2-mm mesh, and stored at 4 °C for further tests.

The three fractions of the EPS (S-EPS, LB-EPS, and TB-EPS) should be separated prior to exploring the compositions and functions of soluble and bound EPS from the activated sludge culture. A combined centrifugation and heating method was used to separate the EPS fractions^6^. According to the literature, this extraction method ensures minimal cell lysis and thus maximally reduces the adverse effects on EPS quantification by unintended release and even dissolution of other substances^13^. The sludge was allowed to settle for 1.5 h and sludge flocs were then washed 3 times with Milli-Q water. Subsequently, the settled flocs were resuspended to a predetermined volume with addition of a buffer solution (pH 7) containing Na_3_PO_4_, NaH_2_PO_4_, NaCl, and KCl at a molar ratio of 2:4:9:1^26^. The suspensions were subsequently stirred for nearly 1 min using a vortex mixer (Maxi Mix II, Thermolyne). Afterward, the suspensions were centrifuged at 2,000 × *g* for 15 min. The supernatant was collected as S-EPS and the remaining centrifuged sediment was again resuspended to the predetermined volume using a warm 0.05% NaCl (w/v) solution (50 °C). Next, the suspensions were centrifuged at 4,000 × *g* for 10 min and the supernatant was collected as LB-EPS. The sediment was again resuspended by addition of a 0.05% NaCl (w/v) solution and heated to 60 °C for 30 min in a water bath. Next, the suspensions were centrifuged at 4,000 × *g* for 20 min, and the organic matter in the supernatant was collected as TB-EPS. The microbial pellet was obtained by resuspending the centrifugation residues. After extraction of each EPS fraction, the particulate matter present in the S-EPS, LB-EPS, and TB-EPS was filtered out using 0.45-μm polytetrafluoroethylene membranes (Shanghai Mosu Scientific Equipment Co., China). Other experimental protocols followed previous work^25^.

Proteins and humic substances were quantified using the modified Lowry method with bovine serum albumin (Sigma-A7030) and humic acids (Sigma-Aldrich-53680) as the standard, respectively^26^. The quantifications of polysaccharides were measured using the anthrone method with glucose (Shanghai Hushi Laboratorial Equipment Co., Ltd, No. 10010518) as the standard^45^.

### Further analysis of EPS extracts

Initially, 3D-EEM fluorescence spectroscopy was applied to identify the fluorescence compounds in the EPS extracts. All extracted EPS fractions were prepared to 1 mg/L in deionized water and their 3D-EEM spectra were determined using a fluorescence spectrophotometer (F-4500, HITACHI, Japan). The 3D-EEM spectra were derived with subsequent scanning emission spectra from 250 nm to 450 nm, at 5-nm increments, by varying the excitation wavelength from 250 nm to 350 nm at 5-nm sampling intervals. The scanning speed was 2,400 nm/min for all measurements. The 3D-EEM data were processed using FL Solutions 2.0 software (HITACHI, Japan).

For the subsequent identification of chemical groups, the FTIR of each EPS was obtained using a FTIR spectrometer (TENSOR 27, Bruker, Germany). The prepared samples were lyophilized and mixed with FTIR-grade KBr power, and OPUS 5.0 operating software was used to generate and process FTIR spectra. Otherwise, the FTIR measurement protocol followed previous work^25^.

### Experimental setup for online aggregation assays

The role of each EPS fraction in the process of sludge aggregation was studied by performing *in situ* aggregation assays before and after extraction of each EPS fraction (i.e., S-EPS, LB-EPS, and TB-EPS). The aggregation assays were carried out using small-angle laser light-scattering (SALS) equipment. Three different sludge samples with bound EPS (i.e., with outer S-EPS removed), with TB-EPS (i.e., with S-EPS and LB-EPS removed), and without EPS (i.e., with all EPS fractions removed and the microbial pellet retained) were added to four reactors (1.0 L each) equipped with agitators, at 500 mg/L, in 0.8 L. In order to simulate the process of microbial aggregation, a stirring speed of 300 rpm (G = 52 s^−1^) was used, following a previous study^46^. The suspensions in each reactor were continuously circulated between the reactor and the SALS equipment, and online observation of floc size was used as a metric for aggregation assays.

### Effects of inter-particle forces on sludge bioaggregation

To date, few studies have reported how to pinpoint key inter-particle forces within an activated sludge matrix; therefore, a typical method used in mechanistic exploration of microbial flocculants was applied in this work. To identify inter-particle forces within microbial flocculants, chemical bond interrupters and/or destroyers are frequently used. For example, EDTA and urea were utilized to identify ion interactions and hydrogen bond, respectively. Previous relevant studies suggested that EDTA is able to chelate metallic ions and inhibits ionic interactions in the medium^47^, whereas urea enables the weakening of electrostatic interactions within proteins, thereby reducing hydrogen bonds^48^,^49^. As the EPS fractions derived from material such as the activated sludge culture studied in this work have similar chemical structures and functions with microbial flocculants, the application of EDTA and urea was carried out to explore the potential links between inter-particle forces and sludge aggregation. Accrodingly, a trial strategy was developed to test the hypothesis that sludge aggregation is mediated by ionic interactions and/or hydrogen bonds that bind microbial cells closely, by adding EDTA and urea to test the two respective cases.

Exploring a suitable procedure to simulate the bioaggregation process is a necessary precursor to identifying the above-mentioned key-particle forces. In our previous work, we found that the original activated sludge floc fails to further aggregate with others, as shown by an insignificant increase (*p* > 0.05) in floc size under favorable stirring conditions (data not shown). According to the literature, sludge flocs can be broken into smaller pieces under high stirring forces^46^. As shown in Figure S1, the average diameter of the sludge floc decreased sharply from 500 μm to less than 60 μm within 1 min at a stirring speed of 1300 rpm (G = 469 s^−1^); however, enhancing the stirring force further did not produce a significant decrease in floc size (*p* > 0.05). Thus, the stirring force of 1300 rpm was chosen to facilitate floc breakage.

As further indicated in Figure S2, the small sludge pellets were re-aggregated accordingly, and the average size of the sludge floc reached its original level (nearly 500 μm) within 5 min at a stirring speed of 300 rpm (G = 52 s^−1^). Therefore, the stirring speed of 300 rpm was selected as the optimal condition to simulate the aggregation process. Consequently, before the addition of EDTA and urea, the aggregated sludge flocs were stirred for 1.5 min in the reactors at a stirring speed of 1300 rpm and broken down into small pellets. Then, the re-aggregation performance of sludge flocs was compared in 6 identical reactors, to which were added 0.1 mol/L, 1.0 mol/L, and 10 mol/L of EDTA and 0.1 mol/L, 1.0 mol/L, and 10 mol/L of urea. A sample to which neither EDTA nor urea was added served as the control. Subsequently, all samples were stirred for 15 min in the reactors at a stirring speed of 300 rpm to simulate the aggregation process. During this step, the floc size was continuously monitored online using the SALS equipment.

### Statistical analysis

The statistical differences between samples were analyzed using IBM SPSS statistics 21.0 software, and *p* < 0.05 was considered statistically significant.

## Additional Information

**How to cite this article**: Guo, X. *et al.* Composition analysis of fractions of extracellular polymeric substances from an activated sludge culture and identification of dominant forces affecting microbial aggregation. *Sci. Rep.* **6**, 28391; doi: 10.1038/srep28391 (2016).

## Supplementary Material

Supplementary Information

## Figures and Tables

**Table 1:** Content and composition of the activated sludge-derived EPS.

Content	EPS composition	Value (mg/g of MLVSS)
S-EPS	PN	4.4
	PS	11.2
	HS	0.6
	Total	16.1
	Ratio of HS, PN, and PS	0.1:0.4:1
	S-EPS (%)	7.4
LB-EPS	PN	18.6
	PS	25.7
	HS	5.1
	Total	49.4
	Ratio of HS, PN, and PS	0.2:0.7:1
	LB-EPS (%)	22.6
TB-EPS	PN	86.7
	PS	50.6
	HS	15.6
	Total	152.9
	Ratio of HS, PS, and PN	0.2:0.6:1
	TB-EPS (%)	70.0
Ratio of S- EPS, LB-EPS, and TB-EPS		0.1:0.3:1

PN-proteins; PS-polysaccharides; HS-humic substances.

Values represent the means of three tests, and the standard deviations were lower than 10% (data not shown).

## References

[b1] Wu Y. H., Li T. L. & Yang L. Z. Mechanisms of removing pollutants from aqueous solutions by microorganisms and their aggregates: A review. Bioresour. Technol. 107, 10–18 (2012). 22257855 10.1016/j.biortech.2011.12.088

[b2] Ding Y., Tian Y., Li Z. P., Zuo W. & Zhang J. A comprehensive study into fouling properties of extracellular polymeric substance (EPS) extracted from bulk sludge and cake sludge in a mesophilic anaerobic membrane bioreactor. Bioresour. Technol. 192, 105–114 (2015). 26022972 10.1016/j.biortech.2015.05.067

[b3] Ding Z. J. *et al.* Role of extracellular polymeric substances (EPS) production in bioaggregation: application to wastewater treatment. Appl. Microbiol. Biot. 99, 9883–9905 (2015). 10.1007/s00253-015-6964-8 26381665

[b4] Tian Y. Behaviour of bacterial extracellular polymeric substances from activated sludge: a review. Int. J. Environ. Pollut. 32, 78–89 (2008).

[b5] Sheng G. P., Yu H. Q. & Li X. Y. Extracellular polymeric substances (EPS) of microbial aggregates in biological wastewater treatment systems: A review. Biotechol. Adv. 28, 882–894 (2010). 10.1016/j.biotechadv.2010.08.001 20705128

[b6] Yu G. H., He P. J. & Shao L. M. Characteristics of extracellular polymeric substances (EPS) fractions from excess sludges and their effects on bioflocculability. Bioresour. Technol. 100, 3193–3198 (2009). 19269815 10.1016/j.biortech.2009.02.009

[b7] Liu X. M. *et al.* Contribution of extracellular polymeric substances (EPS) to the sludge aggregation. Environ. Sci. Technol. 44, 4355–4360 (2010). 20446688 10.1021/es9016766

[b8] Nielsen P. H. & Jahn A. In Microbial extracellular polymeric substances: characterization, structure and function Vol. Chapter 3 (eds Wingender J., Neu T.R. & Flemming H.C.) Ch. 3, 49–72 (Springer-Verlag, 1999).

[b9] Urbain V., Block J. C. & Manem J. Bioflocculation in activated-sludge -An analytic approach. Water Res. 27, 829–838 (1993).

[b10] Badireddy A. R. *et al.* Role of extracellular polymeric substances in bioflocculation of activated sludge microorganisms under glucose-controlled conditions. Water Res. 44, 4505–4516 (2010). 20619438 10.1016/j.watres.2010.06.024

[b11] Wilen B. M., Lumley D., Mattsson A. & Mino T. Relationship between floc composition and flocculation and settling properties studied at a full scale activated sludge plant. Water Res. 42, 4404–4418 (2008). 18752825 10.1016/j.watres.2008.07.033

[b12] Sheng G.-P., Yu H.-Q. & Li X.-Y. Extracellular polymeric substances (EPS) of microbial aggregates in biological wastewater treatment systems: A review. Biotechnol. Adv. 28, 882–894 (2010). 20705128 10.1016/j.biotechadv.2010.08.001

[b13] Li X. Y. & Yang S. F. Influence of loosely bound extracellular polymeric substances (EPS) on the flocculation, sedimentation and dewaterability of activated sludge. Water Res. 41, 1022–1030 (2007). 16952388 10.1016/j.watres.2006.06.037

[b14] Wang B.-B. *et al.* A new classification paradigm of extracellular polymeric substances (EPS) in activated sludge: Separation and characterization of exopolymers between floc level and microcolony level. Water Res. 64, 53–60 (2014). 25043794 10.1016/j.watres.2014.07.003

[b15] Li X. Y. & Yang S. F. Influence of loosely bound extracellular polymeric substances (EPS) on the flocculation, sedimentation and dewaterability of activated sludge. Water Res. 41, 1022–1030 (2007). 16952388 10.1016/j.watres.2006.06.037

[b16] Hermansson M. The DLVO theory in microbial adhesion. Colloids Surfaces B. 14, 105–119 (1999).

[b17] Liao B. Q., Allen D. G., Leppard G. G., Droppo I. G. & Liss S. N. Interparticle interactions affecting the stability of sludge flocs. J. Colloid Interf. Sci. 249, 372–380 (2002). 10.1006/jcis.2002.8305 16290611

[b18] Sobeck D. & Higgins M. Examination of three theories for mechanisms of cation-induced bioflocculation. Water Res. 36, 527–538 (2002). 11827315 10.1016/s0043-1354(01)00254-8

[b19] Wilen B. M., Jin B. & Lant P. The influence of key chemical constituents in activated sludge on surface and flocculating properties. Water Res. 37, 2127–2139 (2003). 12691899 10.1016/S0043-1354(02)00629-2

[b20] Sponza D. T. Investigation of extracellular polymer substances (EPS) and physicochemical properties of different activated sludge flocs under steady-state conditions. Enzyme Microb. Tech. 32, 375–385 (2003).

[b21] Chen W., Westerhoff P., Leenheer J. A. & Booksh K. Fluorescence excitation - Emission matrix regional integration to quantify spectra for dissolved organic matter. Environ. Sci. Technol. 37, 5701–5710 (2003). 14717183 10.1021/es034354c

[b22] Plaza C., Xing B., Fernandez J. M., Senesi N. & Polo A. Binding of polycyclic aromatic hydrocarbons by humic acids formed during composting. Environ. Pollut. 157, 257–263 (2009). 18801605 10.1016/j.envpol.2008.07.016

[b23] Zhu L. *et al.* Specific component comparison of extracellular polymeric substances (EPS) in flocs and granular sludge using EEM and SDS-PAGE. Chemosphere 121, 26–32 (2015). 25441926 10.1016/j.chemosphere.2014.10.053

[b24] Zhang P. *et al.* Composition of EPS fractions from suspended sludge and biofilm and their roles in microbial cell aggregation. Chemosphere 117, 59–65 (2014). 24968163 10.1016/j.chemosphere.2014.05.070

[b25] Ruan X. D., Li L. & Liu J. X. Flocculating characteristic of activated sludge flocs: Interaction between Al^3+^ and extracellular polymeric substances. J. Environ. Sci.-China 25 (2013). 10.1016/s1001-0742(12)60210-1 24218821

[b26] Frolund B., Griebe T. & Nielsen P. H. Enzymatic-activity in the activated-sludge floc matrix. Appl. Microbiol. Biot. 43, 755–761 (1995). 10.1007/BF00164784 7546613

[b27] Kovacs A., Nemcsok D. S. & Kocsis T. Bonding interactions in EDTA complexes. J. Mol. Struc.-Theochem 950, 93–97, doi: 10.1016/j.theochem.2010.03.036 (2010).

[b28] Liu X.-M. *et al.* Contribution of Extracellular Polymeric Substances (EPS) to the Sludge Aggregation. Environ. Sci. Technol. 44, 4355–4360 (2010). 20446688 10.1021/es9016766

[b29] Kelly T. R. & Kim M. H. Relative binding affinity of carboxylate and its isosteres: Nitro, phosphate, phosphonate, sulfonate, and .delta.-lactone. J. Am. Chem. Soc. 116, 7072–7080 (1994).

[b30] Wilox C., Kim E. & Romano D. Experimental and theoretical studies of subsituent effects in hydrogen bond based molecular recognition of a zwicterion by substituted arylurens. Tetrahedron 2, 621–634 (1995).

[b31] Linton B. R., Goodman M. S. & Hamilton A. D. Nitronate anion recognition and modulation of ambident reactivity by hydrogen-Bonding receptors. Chem.-Eur. J. 6, 2449–2455 (2000). 10939746 10.1002/1521-3765(20000703)6:13<2449::aid-chem2449>3.0.co;2-9

[b32] Higgins M. J. & Novak J. T. Characterization of exocellular protein and its role in bioflocculation. J. Environ. Eng.-Asce 123, 479–485 (1997).

[b33] Adav S. S. & Lee D. J. Extraction of extracellular polymeric substances from aerobic granule with compact interior structure. J. Hazard. Mater. 154, 1120–1126 (2008). 18162303 10.1016/j.jhazmat.2007.11.058

[b34] Yuan S. J. *et al.* Identification of key constituents and structure of the extracellular polymeric substances excreted by bacillus megaterium TF10 for their flocculation capacity. Environ. Sci. Technol. 45, 1152–1157 (2011). 21174469 10.1021/es1030905

[b35] Ganesh Kumar C., Joo H. S., Choi J. W., Koo Y. M. & Chang C. S. Purification and characterization of an extracellular polysaccharide from haloalkalophilic Bacillus sp I-450. Enzyme Microb. Tech. 34, 673–681 (2004).

[b36] Struijs J., Stoltenkamp J. & Meent D. V. d. A spreadsheet-based box model to predict the fate of xenobiotics in a municipal wastewater treatment plant. Water Res. 25, 891–900 (1991).

[b37] Ganaye V. A., Keiding K., Vogel T. M., Viriot M. L. & Block J. C. Evaluation of soil organic matter polarity by pyrene fluorescence spectrum variations. Environ. Sci. Technol. 31, 2701–2706 (1997).

[b38] McSwain B. S., Irvine R. L., Hausner M. & Wilderer P. A. Composition and distribution of extracellular polymeric substances in aerobic flocs and granular sludge. Appl. Environ. Microb. 71, 1051–1057 (2005). 10.1128/AEM.71.2.1051-1057.2005 PMC546788 15691965

[b39] Whitchurch C. B., Tolker-Nielsen T., Ragas P. C. & Mattick J. S. Extracellular DNA required for bacterial biofilm formation. Science 295, 1487–1487 (2002). 11859186 10.1126/science.295.5559.1487

[b40] Steinberger R. E. & Holden P. A. Extracellular DNA in single- and multiple-species unsaturated biofilms. Appl. Environ. Microb. 71, 5404–5410 (2005). 10.1128/AEM.71.9.5404-5410.2005 PMC1214645 16151131

[b41] Flemming H. C. & Wingender J. The biofilm matrix. Nat. Rev. Microbiol. 8, 623–633 (2010). 20676145 10.1038/nrmicro2415

[b42] Allesen-Holm M. *et al.* A characterization of DNA release in Pseudomonas aeruginosa cultures and biofilms. Mol. Microbiol. 59, 1114–1128 (2006). 16430688 10.1111/j.1365-2958.2005.05008.x

[b43] Molin S. & Tolker-Nielsen T. Gene transfer occurs with enhanced efficiency in biofilms and induces enhanced stabilisation of the biofilm structure. Curr. Opin. Biotech. 14, 255–261 (2003). 12849777 10.1016/s0958-1669(03)00036-3

[b44] Pellicer-Nacher C., Domingo-Felez C., Mutlu A. G. & Smets B. F. Critical assessment of extracellular polymeric substances extraction methods from mixed culture biomass. Water Res. 47, 5564–5574 (2013). 23866135 10.1016/j.watres.2013.06.026

[b45] Trevelyan W. E., Forrest R. S. & Harrison J. S. Dtermination of yeast carbohydrates with anthrone regent. Nature 170, 626–627 (1952). 13002392 10.1038/170626a0

[b46] Biggs C. A. & Lant P. A. Activated sludge flocculation: On-line determination of floc size and the effect of shear. Water Res. 34, 2542–2550 (2000).

[b47] Garg N. & Garg A. Textbook of Endodontics. 3rd edn, (Jaypee Brothers Medical Publishers (P) Ltd, 2007).

[b48] Mountain R. D. & Thirumalai D. Molecular dynamics simulations of end-to-end contact formation in hydrocarbon chains in water and aqueous urea solution. J. Am. Chem. Soc. 125, 1950–1957 (2003). 12580622 10.1021/ja020496f

[b49] O’Brien E. P., Dima R. I., Brooks B. & Thirumalai D. Interactions between hydrophobic and ionic solutes in aqueous guanidinium chloride and urea solutions: Lessons for protein denaturation mechanism. J. Am. Chem. Soc. 129, 7346–7353 (2007). 17503819 10.1021/ja069232+

