# Supplementary material for: Composition analysis of fractions of extracellular polymeric substances from an activated sludge culture and identification of dominant forces affecting microbial aggregation
Source: Sci Rep. 2016 Jun 17;6:28391. doi: 10.1038/srep28391 (PMC4911604; doi:10.1038/srep28391)
Supplement: Supplementary Information [file srep28391-s1.doc]

**Supplementary Information**

2 figures

**Composition analysis of fractions of extracellular polymeric substances from an activated sludge culture and identification of dominant forces affecting microbial aggregation**

*Xuan Guo ^1,2^,* ^+^*, Xu Wang ^1,3,*^,* ^+^*, Junxin Liu ^1,2,*^*

^1^ Research Center for Eco-Environmental Sciences, Chinese Academy of Sciences, Beijing 100085, China

^2^ University of Chinese Academy of Sciences, Beijing 100049, China

^3^ State Key Laboratory of Aquatic Environmental Chemistry, Research Center for Eco-Environmental Sciences, Chinese Academy of Sciences, Beijing 100085, China

**Corresponding Authors**

^*^ Research Center for Eco-Environmental Sciences, Chinese Academy of Sciences. E-mails: xuwang@rcees.ac.cn (X. Wang); jxliu@rcees.ac.cn (J. Liu)

**Author Contributions**

^+^ X. Guo and X. Wang contributed equally to this work.


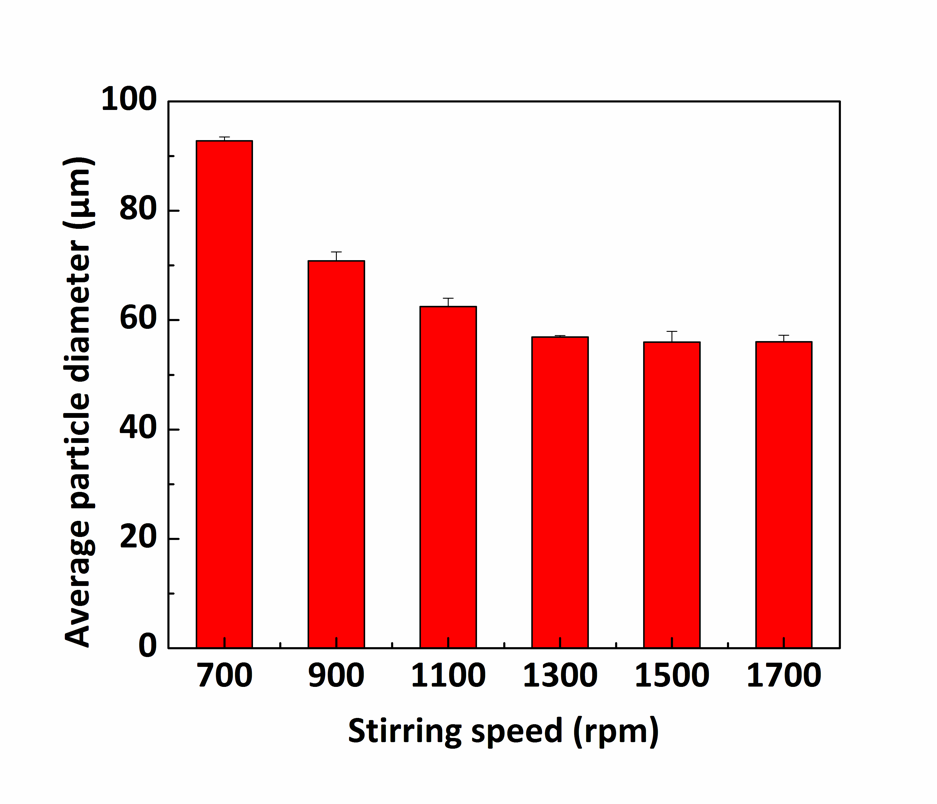


**Figure S1**. Effects of stirring speed on sludge floc breakage, as shown by changes in average particle diameter of sludge floc.


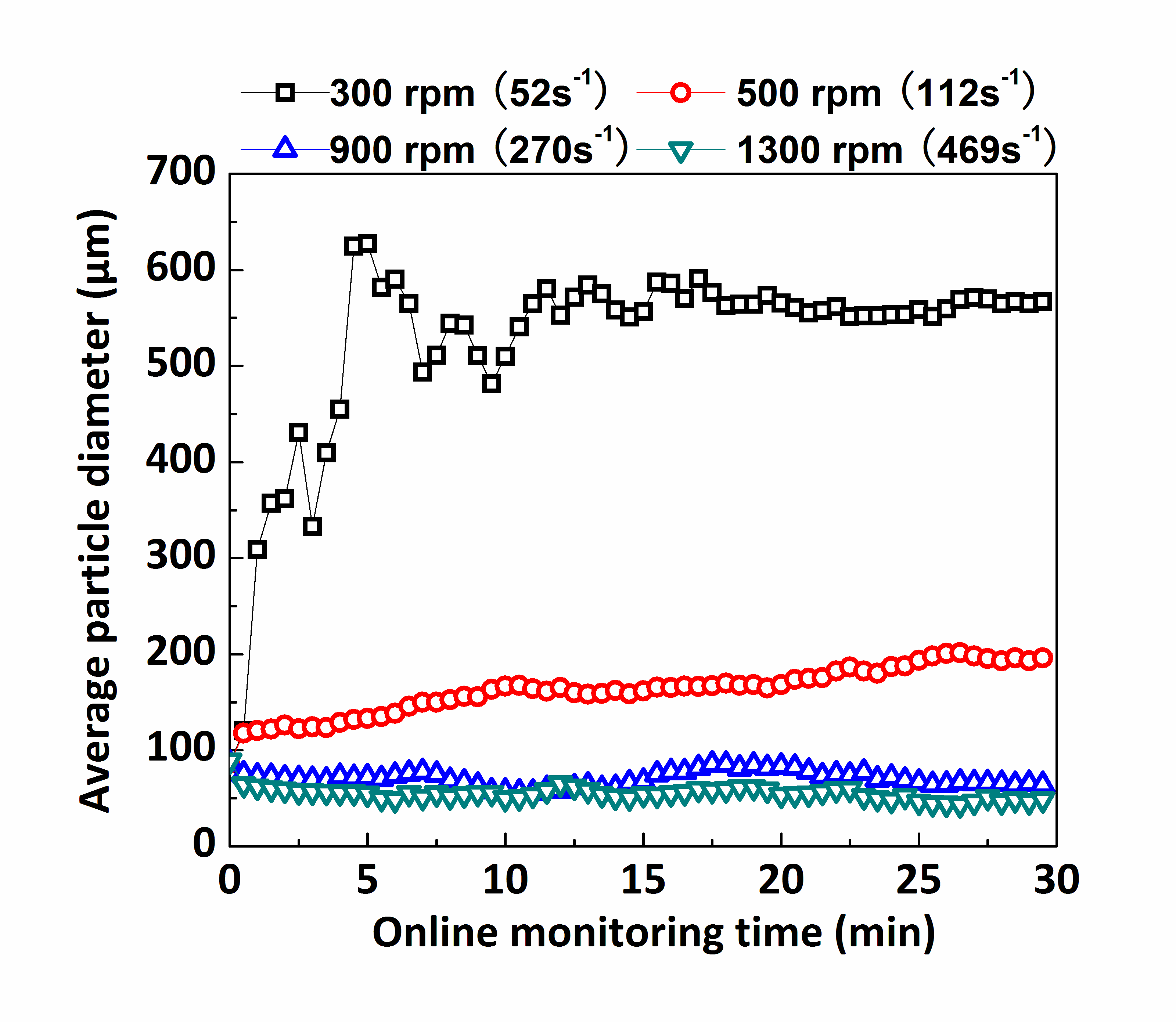


**Figure S2**. Effects of stirring force on re-aggreagation performance of small sludge pellets, as shown by changes in average particle diameter of floc over time.
